# A translational approach for limb vascular delivery of the micro-dystrophin gene without high volume or high pressure for treatment of Duchenne muscular dystrophy

**DOI:** 10.1186/1479-5876-5-45

**Published:** 2007-09-24

**Authors:** Louise R Rodino-Klapac, Paul ML Janssen, Chrystal L Montgomery, Brian D Coley, Louis G Chicoine, K Reed Clark, Jerry R Mendell

**Affiliations:** 1 Center for Gene Therapy, Columbus Children's Research Institute, Columbus Children's Hospital, 700 Children's Dr., Columbus, Ohio, 43205, USA; 2 Department of Pediatrics and Neurology, The Ohio State University, Columbus, OH 43210, USA; 3 Department of Physiology and Cell Biology, 304 Hamilton Hall, College of Medicine, The Ohio State University, Columbus, OH 43210, USA; 4 Department of Radiology, Columbus Children's Hospital, 700 Children's Dr, Columbus, OH 43205, USA

## Abstract

**Background:**

Duchenne muscular dystrophy (DMD) is an X-linked recessive disorder with monogenic mutations setting the stage for successful gene therapy treatment. We have completed a study that directly deals with the following key issues that can be directly adapted to a gene therapy clinical trial using rAAV considering the following criteria: 1) A regional vascular delivery approach that will protect the patient from widespread dissemination of virus; 2) an approach to potentially facilitate safe passage of the virus for efficient skeletal muscle transduction; 3) the use of viral doses to accommodate current limitations imposed by vector production methods; 4) and at the same time, achieve a clinically meaningful outcome by transducing multiple muscles in the lower limb to prolong ambulation.

**Methods:**

The capacity of AAV1, AAV6 or AAV8 to cross the vascular endothelial barrier carrying a micro-dystrophin cDNA was compared under identical conditions with delivery through a catheter placed in the femoral artery of the mdx mouse. Transduction efficiency was assessed by immuno-staining using an antibody (Manex1a) that recognizes the N-terminus of micro-dystrophin. The degree of physiologic correction was assessed by measuring tetanic force and protection from eccentric contraction in the extensor digitorum longus muscle (EDL). The vascular delivery paradigm found successful in the mouse was carried to the non-human primate to test its potential translation to boys with DMD.

**Results:**

Regional vascular delivery resulted in transduction by rAAV8.micro-dystrophin reaching 94.5 ± 0.9 (1 month), 91.3 ± 3.1 (2 months), and 89.6 ± 1.6% (3 months). rAAV6.micro-dystrophin treated animals demonstrated 87.7 ± 6.8 (1 month), 78.9 ± 7.4 (2 months), and 81.2 ± 6.2% (3 months) transduction. In striking contrast, rAAV1 demonstrated very low transduction efficiency [0.9 ± 0.3 (1 month), 2.1 ± 0.8 (2 months), and 2.1 ± 0.7% (3 months)] by vascular delivery. Micro-dystrophin delivered by rAAV8 and rAAV6 through the femoral artery significantly improved tetanic force and protected against eccentric contraction. Mouse studies translated to the hindlimb of cynamologous macaques using a similar vascular delivery paradigm. rAAV8 carrying eGFP in doses proportional to the mouse (5 × 10^12 ^vg/kg in mouse vs 2 × 10^12 ^vg/kg in monkey) demonstrated widespread gene expression [medial gastrocnemius – 63.8 ± 4.9%, lateral gastrocnemius – 66.0 ± 4.5%, EDL – 80.2 ± 3.1%, soleus – 86.4 ± 1.9%, TA – 72.2 ± 4.0%.

**Conclusion:**

These studies demonstrate regional vascular gene delivery with AAV serotype(s) in mouse and non-human primate at doses, pressures and volumes applicable for clinical trials in children with DMD.

## Background

Duchenne muscular dystrophy (DMD) is the most common, genetically inherited, progressive muscle disease of childhood affecting 1 in 3,500 newborn males. [1] A limited treatment option can be offered through the use of corticosteroids, prolonging ambulation in some but not all patients. [2] Multiple treatment strategies are in evolution including some currently being tested in clinical trials. These include exon skipping [3,4], mutation suppression for premature stop codons [5,6], and pharmacologic agents promoting muscle growth modulation. [7,8] Promising findings are also emerging from experimental gene replacement therapy in dystrophic mice [9-12] and dogs. [4,13,14] In the clinic, lessons learned from these studies are currently restricted to recombinant AAV (rAAV) mediated gene delivery to single muscles by direct injection. While this approach is important in laying the foundation for future studies, clinically meaningful results would best be attained through gene transfer reaching widespread muscle targets in order to significantly improve the quality of life for an individual patient. Gene delivery via a vascular route offers this possibility, dictating a direction to be followed in planning future clinical trials.

In the mouse, AAV serotypes 6 and 8 have been shown to be well-suited for systemic vascular delivery. Gregorevic et al. illustrated widespread vascular delivery of the micro-dystrophin cassette to skeletal muscle in the mdx mouse [9,10], while Wang et al. demonstrated the potential for rAAV8 vectors to achieve skeletal muscle transduction in limb muscles remote from the delivery site. [15] These prior studies are of interest to clinical trialists but do not translate directly to the bedside. The very large viral load required to achieve widespread AAV6 transduction using tail vein delivery in the mdx mouse would challenge current vector production methods, clearly limiting clinical application. The AAV8 studies employed neonatal mice, where vascular permeability factors differ from the more mature vascular bed of the DMD patient. In addition, AAV1 cannot be excluded from consideration because of its established efficiency for skeletal muscle transduction following direct injection [16,17] and its demonstrated potential for robust dystrophin expression following vascular delivery through the femoral artery of a specialized exon skip cassette in the mdx model. [18] However, the true potential for adapting AAV1 to a vascular delivery clinical trial is unknown because the mouse studies employed a large fluid volume, rapidly delivered through the femoral artery, accompanied by rupture the capillary endothelial bed and edema of the lower limb extremities.

While these observations in the mouse are important, none of the studies meet the following criteria that can be directly adapted to a clinical trial:

1) A regional vascular delivery approach that protects the patient from widespread dissemination of virus; 2) permits safe passage of the virus given more recent information on the immunogenicity of AAV [19]; 3) uses viral doses that will accommodate current limitations imposed by vector production methods; and at the same time, 4) achieves a clinically meaningful outcome (e.g., prolonged ambulation by transducing multiple muscles in the limb). The study presented here attempts to meet these criteria. The first step toward achieving our goal was to test AAV1, AAV6 and AAV8 under identical conditions, an experiment not previously done, to establish which serotype is most likely to be successful in a regional vascular delivery clinical trial. Attempting to avoid the trap of translating results directly from the mouse to the patient under the assumption that such studies necessarily predict efficacy at the bedside, we extended our observations to non-human primates employing conditions that would closely simulate a clinical delivery model. The results of this stepwise approach are presented here.

## Methods

### Animals and Treatments

All procedures were approved by the CCRI Institutional Animal Care and Use Committee. Three to four week old mdx mice and normal age-matched C57/BL10 were used for vascular delivery and force generation studies of the extensor digitorum longus (EDL) muscle. Two-year old cynamologous macaques were pre-screened for binding antibodies to AAV8 by ELISA and those that scored negative at a 1:50 serum dilution were included for isolated limb perfusion studies. All non-human primates were housed in pairs to promote socialization.

### Isolated Limb Perfusion Studies in the Mouse

Mice were weighed and then anesthetized with a combination of ketamine and xylazine (100 mg/kg and 10 mg/kg, respectively). The left groin was shaved to the mid-thigh and treated with 95% ethanol and Povidine iodine solution. The femoral bundle was visualized via a small incision proximal to the mid-thigh and vessels were dissected away from the femoral nerve. A 6-0 braided silk tourniquet was placed around the limb under the vessel, such that, when tightened, blood flow in and out of the leg was prevented. Blood flow through the femoral artery and vein was controlled by catheter placement in each vessel using a customized heat pulled polypropylene 10 (PE-10) catheter placed into the vessels using an introducer hole with a 33-gauge needle. Prior to vector administration, the arterial catheter was flushed (pre-flush) with 100 μl sterile normal saline. Immediately prior to vector administration all blood flow to the extremity was impeded (isolated limb perfusion – ILP) by tightening the ligature at the mid-thigh. The femoral vein catheter was also occluded preventing vascular egress from the extremity. A dose of 1 × 10^11 ^vg (rAAV1, rAAV6, or rAAV8) was perfused through the femoral artery in 100 μl of sterile Tris buffered saline administered at a rate of approximately 2 μl per second (over 60–80 seconds). After 10 minutes of maintained vascular occlusion, the femoral venous catheter was opened and 100 ul of normal saline was administered to the arterial catheter (again at about 2 μl per second) as a post-flush. Non-tissue bound vector exited the extremity through the patent venous side. The mid-thigh tourniquet was then released, the catheters removed, and bleeding was controlled by direct pressure to the vessels. The wound was flushed with sterile normal saline and closed with a 6-0 proline suture.

For intramuscular injections, mice were anesthetized and maintained on 1–4% Isoflurane (in Oxygen). Both hind limbs were shaved and the tibialis anterior muscle was injected with 3 × 10^10 ^vg of rAAV1, 6, or 8.micro-dystrophin or normal saline (30 μl volume) using a 30 gauge ultra-fine insulin syringe.

### Isolated Limb Perfusion Studies in Macaques

Cynamologous macaques were sedated with intramuscular telazol (3–6 mg/kg), intubated and secured to a heated procedure table at 37°C. General anesthesia was administered with Isoflurane (in Oxygen) 1–4% during the procedure. The left groin was shaved with extension to the mid thigh, and prepped with 95% ethanol and Povidine iodine solution. Working with a larger animal (2–3 Kg) has advantages for catheter placement and more closely simulates the leg of a child. A groin incision was made and the femoral artery and vein were isolated. The femoral artery was catheterized using a custom made, fluoroscopy guided 3-0 f catheter that was advanced to the arterial bifurcation at the level of the knee. Prior to vector administration a pre-vector flush was performed using a 2 ml volume of saline. This was immediately followed by occluding blood flow to the extremity using a standard phlebotomy tourniquet placed above the exposed femoral artery and vein. For these studies, only a single vector construct, rAAV8.CMV.eGFP, was infused over 60 seconds at a dose of 10^13 ^vg (2 × 10^12^/kg) in 2 ml of Tris buffered saline. The extremity remained isolated from the circulation for 10 minutes before releasing the tourniquet. A post-vector flush consisting of a volume of 2 ml was infused simultaneous to the release of the thigh tourniquet. Hemostasis and wound closure were similar to the mouse experiments.

### Micro-dystrophin gene construction

The murine micro-dystrophin construct possessed the (R4–R23/Δ71–78) domains as previously described. [20] The cDNA was codon optimized for rodents and synthesized by GenScript Inc. It includes a consensus Kozak sequence, an SV40 intron, and synthetic polyadenylation site (53 bp). A truncated MCK (muscle creatine kinase) promoter/enhancer was used to drive muscle specific gene expression. The MCK micro-dystrophin expression cassette was cloned between AAV2 ITRs using flanking Xba I restriction enzyme sites in plasmid derived from pCMVβ (Clontech). Msc I/Sma I restriction enzyme digestions were used to confirm ITR integrity.

### rAAV Vector production

rAAV vectors were produced by a modified cross-packaging approach whereby the AAV type 2 vector genome can be packaged into multiple AAV capsid serotypes. [21] Production was accomplished using a standard 3 plasmid DNA/CaPO_4 _precipitation method using HEK293 cells. 293 cells were maintained in DMEM supplemented with 10% fetal bovine serum (FBS) and penicillin and streptomycin. The production plasmids were: (i) pAAV.MCK.microdys, (ii) rep2-capX modified AAV helper plasmids encoding cap serotypes 1, 6, or an 8-like isolate, and (iii) an adenovirus type 5 helper plasmid (pAdhelper) expressing adenovirus E2A, E4 ORF6, and VA I/II RNA genes. To allow comparisons between serotypes, a quantitative PCR-based titration method was used to determine an encapsidated vector genome (vg) titer utilizing a Prism 7500 Taqman detector system (PE Applied Biosystems). [22] The primer and fluorescent probe targeted the MCK promoter and were as follows: MCK forward primer, 5-CCCGAGATGCCTGGTTATAATT-3; MCK reverse primer, 5-GCTCAGGCAGCAGGTGTTG-3; and MCK probe, 5-FAM-CCAGACATGTGGCTGCTCCCCC-TAMRA-3.

### Gene Expression analysis

Tibialis anterior (TA) and gastrocnemius skeletal muscles were collected from mdx treated and contralateral control limbs at 4, 8, and 12 weeks post treatment. Muscles were embedded in 7% gum tragacanth and flash frozen in isopentane cooled in liquid nitrogen. Cryostat sections (12 μm) for immunostains were incubated with the N-terminal Manex1a primary antibody (Developmental Studies Hybridoma Bank), alpha-sarcoglycan (Vector, VP-A108), or beta-sarcoglycan (Vector, VP-B206) primary antibody at a dilution of 1:50 in blocking buffer (PBS, 10% goat serum, 0.1% Triton x-100) for 1 hour at room temperature in a wet chamber. Sections were then washed with PBS three times, each for 20 minutes and re-blocked. Visualization was achieved by incubation for 30 minutes at room temperature with an Alexa 488 or Alexa 594-conjugated isotype-specific goat anti-mouse antibody at a 1:300 dilution (Molecular Probes). Sections were washed in PBS 3 times for 20 minutes and mounted with Vectashield mounting medium (Vector Laboratories). Fluorescence staining for micro-dystrophin was visualized at an excitation wavelength of 488 nm for fluorescein isothiocyanate (FITC) using a Zeiss Axioskop2 Plus Microscope and images were captured with a Zeiss AxioCam MRC5 camera. The number of fibers with sarcolemmal dystrophin staining was expressed as percent of all dystrophin positive fibers.

### Western Blot Analysis

Tissue sections from wild-type C57/BL10, mdx rAAV.micro-dystrophin treated, and mdx control mice (10–20 μm thick) were collected into a microcentrifuge tube and homogenized with 100 μl homogenization buffer (125 mM Tris-HCL, 4% SDS, 4 M urea, 5% β-mercaptoethanol, 10% glycerol, 0.001% bromophenol blue, 1 N HCL) in the presence of 1 protease inhibitor cocktail tablet (Roche). After homogenization, the samples were boiled for 5 min and centrifuged at 16,000 × g for 5 minutes. The total protein content of the supernatant was measured using the Bio-Rad Protein Assay (Bio-Rad Laboratories). Protein samples (75 μg per lane) were electrophoresed on a 3–8% polyacrylamide Tris-Acetate gel (NuPAGE, Invitrogen) for 16 h at 23 V and then transferred to a PVDF membrane (Amersham Biosciences) for 1 h at 100 V at 4°C. The membrane was blocked in 5% nonfat dry milk in TBST (100 mM Tris-HCL, pH 8.0, 167 mM NaCL, 0.1% Tween) for 1 h, and then incubated with a 1:200 dilution of the primary monoclonal antibody Manex1a (Developmental Studies Hybridoma Bank) and a muscle specific actin monoclonal antibody (Vector Labs) in TBST/5% dry milk for 1 h at room temperature in a heat sealed bag. Anti-mouse secondary-HRP (Dako) was used for ECL immunodetection (Amersham Biosciences).

### Morphometrics

Centralized nuclei counts were performed on sections of TA muscles stained with hematoxylin and eosin (H&E) from 3-month-old mdx animals treated with either rAAV6 or rAAV8 micro-dystrophin. TAs from the contralateral extremity served as controls. Five random 20× fields of 12 μm sections for each muscle were captured and the number of fibers with central nuclei counted. Fiber diameter measurements were also performed on TA muscles from 3-month-old mdx animals treated with either rAAV6 or rAAV8 micro-dystrophin and stained with succinic deyhydrogenase to delineate mitochondria-enriched fibers representing slow twitch oxidative (type 1), fast twitch oxidative glycolytic (type 2A), and fast twitch glycolytic (type 2B) fiber types. Controls were contralateral TA muscles. Five random 20× fields of 12 μm sections for each muscle were captured with a Zeiss AxioCam MRC5 camera. For each fiber type, the smallest diameter was measured using Zeiss Axiovision LE4 software. A frequency distribution was performed to represent the percent number of fibers within 10 μm intervals.

### Force generation and protection from eccentric contractions

Eight weeks post gene transfer, mice were euthanized and the extensor digitorum longus (EDL) muscle was removed, weighed, and bathed in oxygenated Krebs-Henseleit solution (95% O_2_/5% CO_2 _(pH 7.4), 118 mM NaCl, 25 mM NaHCO_3_, 5 mM KCl, 1 mM KH_2_PO_4_, 2.5 mM CaCl_2_, 1 mM MgCl_2_, 5 mM Glucose) at 30°C in a circulating bath. One end of the muscle was tied to a force transducer and the other to a high-speed linear servo-controlled motor. Muscle length was changed at predetermined values and speeds using customized software. The muscle was mounted in the set-up at slack length with a resting tension of 1 g for 10 minutes without electrical stimulation. Stimulation was delivered via two parallel platinum-iridium electrodes on either side of the muscle. Muscles were adjusted to optimum length (L0), defined as the length for maximal twitch and subjected to three isometric tetani of 150 Hz, each for 500 ms with 1 minute between stimulations. Following a 5 minute rest period, muscles were subjected to a protocol of a series of 4 isometric tetani with increasing frequencies of 50, 80, 120, and 150 Hz, each for 500 ms with 1 minute between stimulations. Following a 10 minute rest period, muscles were subjected to an eccentric contraction protocol consisting of a series of 10 isometric 700 ms tetani, at 2 minute intervals, with a 10% lengthening of the muscles (0.5 fiber length per second for duration of 200 ms) when maximal force has developed at 500 ms. After the tetanus ended (at t = 700 ms), the muscle was brought back to initial length (at the same speed as the stretch), allowing for full relaxation to the initial length. For comparative purposes, all force measurements are expressed per unit cross-sectional area (normalized isometric force or tension: mN/mm^2^). Cross-sectional area (CSA) was calculated using the following equation, CSA = (muscle mass in g)/[(optimal fiber length in cm) × (muscle density in g/cm^3^)], where muscle density is 1.06 g/cm^3^.

### Statistical analyses

Data were analyzed with standard statistical methods and expressed as means ± SEM (or as indicated). A paired t-test or a one-way ANOVA was used to determine statistical significance between groups. Significance level was set at p = 0.05.

## Results

### Construction of rAAV1, 6, and 8 vectors carrying a micro-dystrophin cDNA

**Figure 1:**
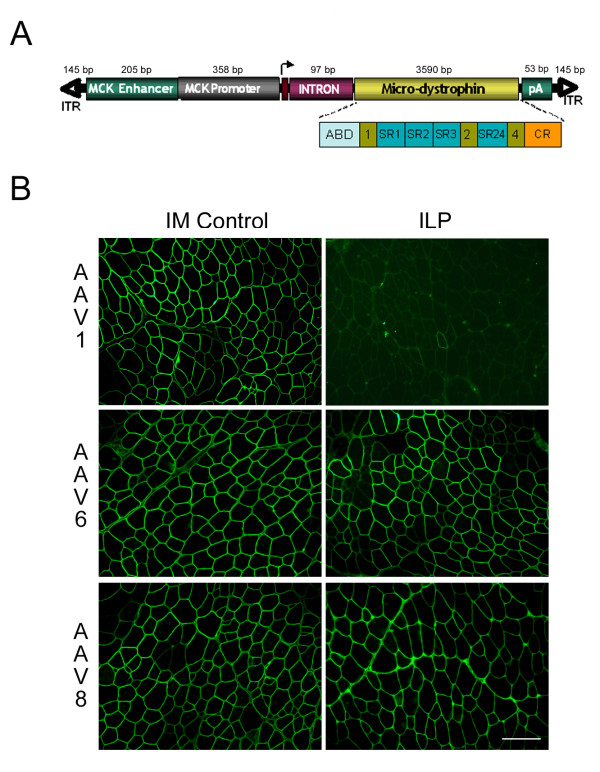
(A) Schematic of murine micro-dystrophin construct. A truncated MCK promoter/enhancer (563 bp) is used to drive muscle specific gene expression. Also labeled is a chimeric SV40 intron (97 bp) and synthetic polyadenylation site (53 bp). The 3,590 bp murine micro-dystrophin construction is depicted in detail. ABD is the complete actin binding domain, hinges 1, 2 and 4 are shown (green boxes), as are the flanking spectrin rod domains (SR blue boxes). The cysteine-rich dystroglycan binding domain is denoted by an orange box. AAV2 ITR are shown as arrowheads. (B) Immunofluorescence detection of micro-dystrophin expression in *mdx *mouse TA muscle. rAAV1, 6, or 8.micro-dystrophin (10^11 ^vg) was delivered by intramuscular injection (IM Control) or ILP through the femoral artery of 3–4 week old *mdx *mice. Representative TA muscle sections (12 um) are shown from 4 week post-injected animals (8 and 12 weeks looked similar). Sections were immuno-stained with the N-terminal dystrophin antibody Manex1a. Scale Bar, 50 μm.

The first goal was the identification of an optimal rAAV serotype demonstrating efficient vascular egress into underlying muscle tissue considering scalability of viral doses, and the use of volumes and perfusion pressures appropriate for clinical application. Based on published work, AAV serotypes 1, 6 and 8, emerged as leading candidates. The expression cassette consisted of a micro-dystrophin gene under the control of a truncated MCK promoter/enhancer (563 bp) to achieve muscle specific gene expression, and avoid promiscuous transgene expression in undesired cell types, helping to reduce transgene-related host immune responses. The micro-dystrophin construct, (R4–R23/Δ71–78), identical to that described by Harper et al. [20] and later used by Yoshimura et al. [12], contains the N-terminus, the actin-binding domain, four spectrin repeats and hinges 1, 2 and 4, and the cysteine-rich domain (Fig. 1A). Murine micro-dystrophin cDNA was synthesized using species specific codon optimization. Several c*is *modifications were added to achieve maximum gene expression: (1) an intron derived from SV40 (120 bp) to augment mRNA processing, stability and nuclear export [23]; (2) an small synthetic polyadenylation signal to minimize construct size and (3) inclusion of a consensus Kozak sequence (CCACC) as a translation initiation signal. All vectors were prepared using an adenovirus-free transient plasmid DNA transfection protocol and were purified by a combination of iodixanol density gradient and fast protein liquid chromatography (FPLC) ion exchange chromatography. Vector genome titers were measured by Taqman Q-PCR as previously described by our group. [22]

### ILP delivery of rAAV6 and rAAV8 results in efficient micro-dystrophin in the mouse hindlimb

**Figure 2:**
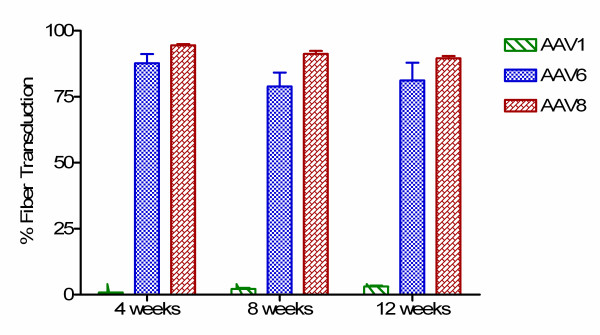
Quantification of micro-dystrophin protein expression. Micro-dystrophin distribution in muscle sections (4, 8, 12) weeks was quantified by visual fiber counts (images from 4 random fields) of the number of positive fibers/total fibers and represented as a percent mean ± SEM (n = 4–8 per group).

rAAV1, 6, or 8.MCK.micro-dystrophin vector was perfused (100 μl volume) at a dose of 10^11 ^vg in sterile Tris buffered saline into the femoral artery of the left hindlimb of 3–4 week old mdx mice. Details of catheter placement are provided in the methods, but certain steps deserve emphasis. Anatomical restrictions in the mouse led to catheter placement below the take off of the deep femoral artery, a major source of blood supply to the thigh and muscle. Prior to vector administration, and before isolating blood flow to the extremity, the arterial catheter was flushed (pre-flush) with 100 μl sterile normal saline. Subsequently a 6-0 braided silk ligature was tightened to prevent all blood flow to the extremity (isolated limb perfusion-ILP) proximal to the site of catheter introduction. Immediately after ligature tightening, vector was slowly administered (over 60 to 80 seconds) to minimize capillary rupture. Following a ten minute dwell time, a 100 ul saline post-flush was performed immediately before removing the tourniquet. The tibialis anterior (TA), extensor digitorum longus (EDL), gastrocnemius, and soleus were harvested at 1, 2, and 3 months post transfer. Micro-dystrophin gene expression was quantified by immuno-staining of muscle cross sections using a monoclonal antibody (Manex-1a) against the N-terminus of micro-dystrophin. Widespread micro-dystrophin expression was observed in the TA and EDL muscles of mice receiving rAAV6 and rAAV8 at all three time points (Fig. 1B). Fiber counts revealed that rAAV8.micro-dystrophin cohorts exhibited 94.5 ± 0.9% (1 month), 91.3 ± 3.1% (2 months), and 89.6 ± 1.6% (3 months) transduction; and rAAV6.micro-dystrophin treated animals demonstrated 87.7 ± 6.8% (1 month), 78.9 ± 7.4% (2 months), and 81.2 ± 6.2% (3 months) transduction (Fig. 2). The gastrocnemius and soleus demonstrated lower levels of expression with mean transduction efficiencies over all 3 time points of 6.7 ± 0.7% for rAAV8.micro-dystrophin and 17.6 ± 1.4% for rAAV6.micro-dystrophin. This is likely attributed to catheter placement and the anatomy of the vasculature of the mouse hindlimb. In the mouse, the femoral artery bifurcates at the level of the knee with one arterial branch supplying the TA and EDL and two smaller branches which turn posteriorly and perfuse the two heads of the gastrocnemius and soleus. The femoral catheter was placed just above the knee with delivery of vector directed toward the more accessible arterial branch to the TA, complicit with a low volume/pressure system. In contrast to rAAV8 and rAAV6, vascular delivery of micro-dystrophin by rAAV1 did not efficiently transduce the muscles of the lower limb with 0.9 ± 0.3% (1 month), 2.1 ± 0.8% (2 months), and 2.1 ± 0.7% (3 months) transduction (Fig. 2). This could not be attributed to the biological activity of the vector preparation since direct muscle injection into the TA, performed in parallel with the vascular injections, resulted in robust and equivalent gene expression for all three serotypes at 1 month post injection; 94.0 ± 1.2% (rAAV1), 95.5 ± 0.7% (rAAV6), and 95.1 ± 0.6% (rAAV8) (Fig. 1B). Our inability to observe vascular delivery using this rAAV1 serotype is in contrast to previous studies using arterial perfusion in mice. [18,24,25] Differences in dose, injection volume, and pressure best explain the dissimilar results (see discussion). Consistent with our immunofluorescence data, western blot analysis revealed the presence of the expected 138 kDa micro-dystrophin protein rAAV6 and 8 cohorts (data not shown).

### Micro-dystrophin expression levels result in reduced mdx pathology

**Figure 3:**
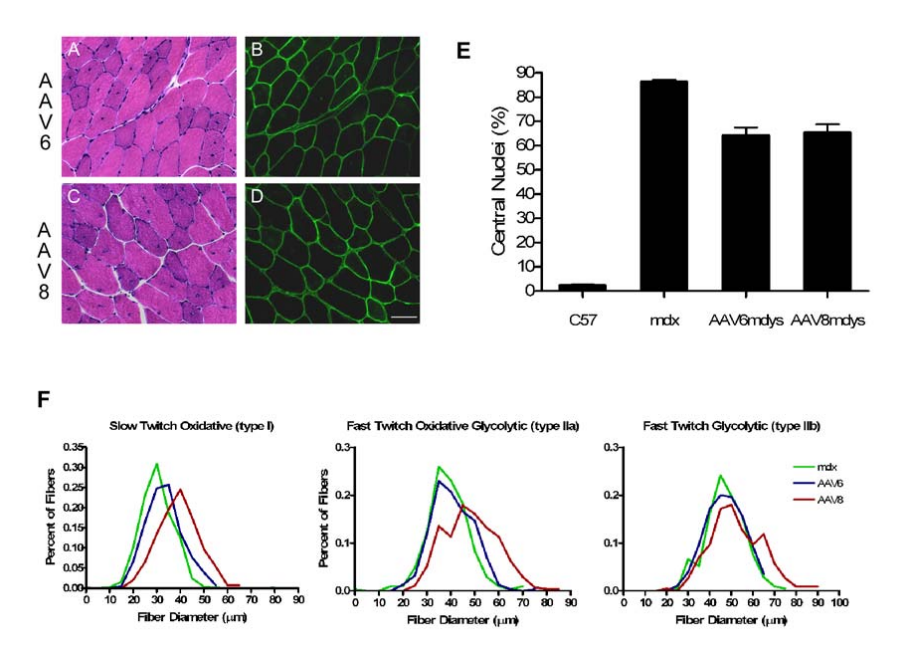
Micro-dystrophin expression levels result in reduced mdx pathology. Hematoxylin and Eosin (H&E) analysis revealed a reduction in centralized nuclei, a hallmark of DMD pathology. (A, C) H&E staining of rAAV6 and rAAV8 micro-dystrophin muscle sections, respectively (8 week time point). (B,D) Micro-dystrophin immunofluorescence detection in serial sections of rAAV6 and rAAV8 cohorts, respectively. Scale Bar, 25 μm. (E) Micro-dystrophin expression results in a 25% decrease in centralized nuclei. (F) Frequency distribution demonstrating micro-dystrophin expression results in a significant increase in type I, IIa, and IIb fiber diameter for rAAV6 and rAAV8.micro-dystrophin injected mdx mice compared to *mdx *controls. (n = 5–6 per group) (p < 0.0001 ANOVA analysis).

**Figure 4:**
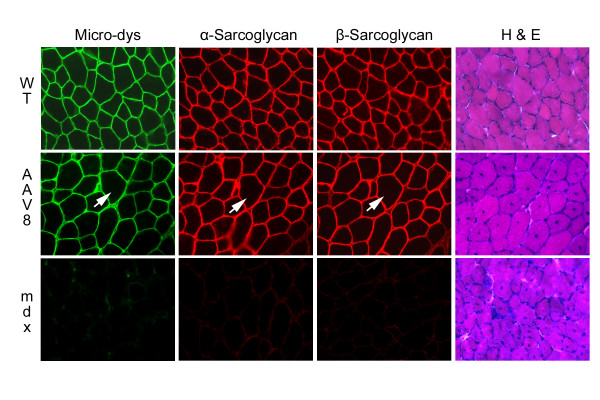
Components of the dystrophin-associated protein complex are restored in *mdx *mice treated with rAAV.micro-dystrophin gene transfer. *mdx *mice treated with rAAV8.micro-dystrophin delivered by ILP (8 week time point) were serial sectioned and stained with manex1a antibody for dystrophin, α-sarcoglycan, β-sarcoglycan, and H &E. Muscle fibers transduced with micro-dystrophin also exhibited restoration of expression of α-sarcoglycan and β-sarcoglycan (no staining in untreated *mdx *mice – bottom row). Muscle fibers from serial sections exhibited identical expression localization for each protein (arrow).

The major wave of necrosis and regeneration in the mdx mouse occurs at approximately 3 to 4 weeks of age, coincident with our vector administration. Maximum gene expression from single-stranded rAAV vectors does not occur until about 4 weeks post gene transfer. Thus, the effects on mdx pathology may be modest. Quantitation of central nuclei was determined in the TA muscles treated with rAAV6 or rAAV8 micro-dystrophin and compared with controls. Central nucleation was reduced by only 25% (rAAV8 – 65.3 ± 3.3%, rAAV6 – 64.05 ± 3.4%, vs mdx – 86.16 ± 0.9%; P < 0.0001; Fig. 3E), reflecting protection against recurring cycles of regeneration and degeneration only between the time of gene expression and necropsy (2 months post gene transfer). Detailed morphometric analysis of fiber diameter revealed that both rAAV6 and rAAV8 treated groups exhibited an increase in myofiber diameter compared to controls. Cross sections from treated and control muscles were stained to delineate slow twitch (type 1), fast twitch oxidative glycolytic (type 2A) and fast twitch glycolytic (type 2B) fiber types, with slow twitch fibers demonstrating the largest increase (Fig. 3F). Significant differences in mean fiber diameter (μm) were seen in rAAV8.microdystrophin treated animals compared to mdx for all three fiber types [type 1–39.2 ± 0.4 (rAAV8) vs 30.2 μm (mdx); type 2A – 48.2 ± 0.7 vs 38.9 ± 0.5 μm; type 2B – 52.4 ± 0.8 vs 47.1 ± 0.6 μm where P < 0.0001], while AAV6.microdystrophin treated animals exhibited significant differences for type 1 (33.2 ± 0.3 μm; P < 0.0001) and type 2A (40.8 ± 0.5 μm; P < 0.01) fibers. The largest differences for both rAAV8 and rAAV6.microdystrophin treated animals were demonstrated in type 1 (slow twitch) fibers with a 23% and 10% increase in fiber diameter, respectively. These differences are further demonstrated in Figure 3 where frequency distribution of fiber diameters stratified by fiber type demonstrates an overall shift toward larger diameter for both rAAV8 and rAAV6.micro-dystrophin cohorts. ANOVA was performed on all data sets and demonstrated significance for both groups compared to mdx controls (P < 0.0001). Lastly, consistent with biological activity, micro-dystrophin levels were sufficient to restore proper localization of dystrophin-associated proteins (DAP) proteins α-sarcoglycan, and β-sarcoglycan in rAAV6 and rAAV8 cohorts (Fig. 4).

### rAAV mediated micro-dystrophin expression via ILP results in significant force generation improvement in mdx animals

**Figure 5:**
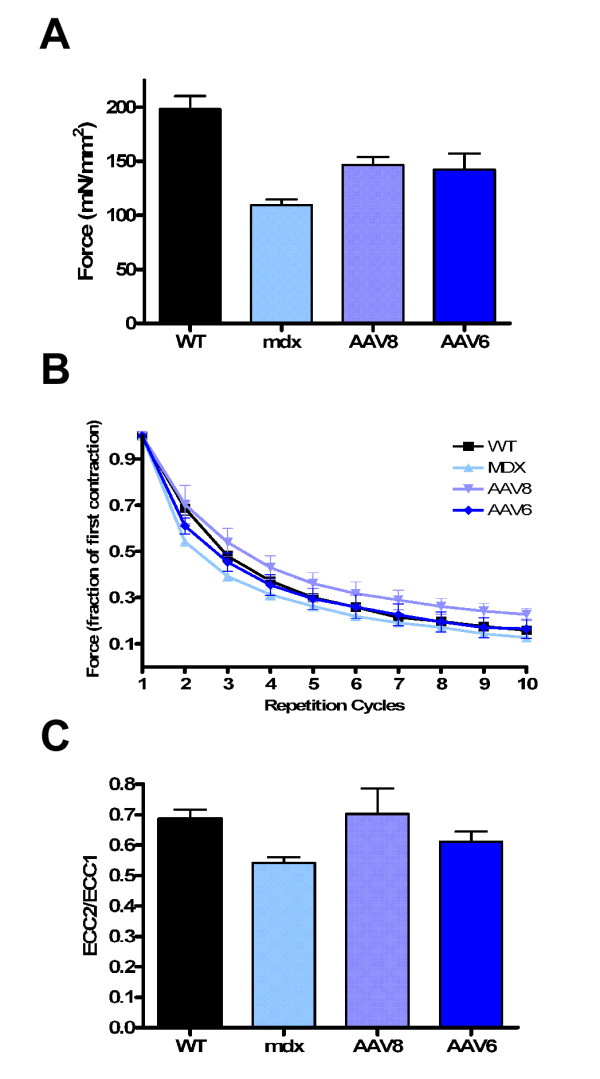
rAAV.micro-dystrophin gene transfer improves force generation measurements significantly. (A) rAAV8.micro-dystrophin and rAAV6.micro-dystrophin treated mdx extensor digitorum longus (EDL) muscles (8 week time point) demonstrated significant maximum specific force improvement (mN/mm^2^) over mdx-untreated muscles (p < 0.05) but did not reach levels of age-matched C57BL/10 wild type (WT) controls (P < 0.05). Values are presented as the means ± SEM, (B) EDL muscles were subjected to 10 cycles of isometric stimulation at 150 Hz with a 10% lengthening to induce damage during the last 100 ms of each contraction. Force values are represented as fractions of the first contraction. rAAV8.micro-dystrophin and rAAV6.micro-dystrophin treated EDL muscles exhibited significant protection from damage compared to mdx-untreated during the first two cycles (p < 0.05), while thereafter all 4 groups showed such robust injury that differences could no longer be determined. There was no significant difference between rAAV8- or rAAV6-treated and WT. (C) The ratio of force (ECC2/ECC1) after one lengthening contraction of rAAV-treated versus the initial contraction of WT is not different, but is significantly improved compared to mdx-untreated (n = 6–8 per group; P < 0.05).

To test whether widespread micro-dystrophin expression led to improvement at a functional level, the force generation properties of the EDL muscle from mdx mice treated with rAAV.micro-dystrophin were compared with untreated mdx and wild-type C57/B10 controls. Eight weeks post transfer, animals were euthanized and the EDL was dissected for in vitro force measurements. rAAV8- and rAAV6-treated muscles exhibited a 50% improvement in maximum isometric force compared to mdx when normalized for the cross-sectional area of the muscle (rAAV8.micro-dystrophin 146.5 ± 7.6 mN/mm^2^, rAAV6.micro-dystrophin 142.2 ± 14.8 mN/mm^2 ^vs. mdx-untreated 109.5 ± 5.1 mN/mm^2^; P < 0.05) (Fig. 5A). The increase in developed force did not completely reach the level of age-matched C57BL/10 wild-type controls (198.3 ± 11.9 mN/mm^2^; P < 0.05) (Fig. 5A). After assessment of specific force, the muscles were subjected to mechanical damage by repetitive eccentric contractions. Micro-dystrophin expression by rAAV8 and rAAV6 showed protection against eccentric contraction injury. Analyzing the force generation after the first lengthening contraction by comparing the ratio of the second versus the first contraction revealed that mdx-untreated muscles decayed 0.54 ± 0.02 versus rAAV8 at 0.70 ± 0.08, and rAAV6 at 0.61 ± 0.04. The treated muscles were significantly more resistant to contraction-induced injury compared to untreated mdx (p < 0.05); neither treated group differed significantly from WT controls, which decay by 0.69 ± 0.08 (Fig. 5B, 5C). Due to the robust eccentric injury protocol, even in WT muscles, from the third contraction on no differences were observed between any groups. Combined, these data demonstrate that micro-dystrophin delivered by either rAAV6 or rAAV8 through the vasculature leads to significant improvement in isometric developed force and significantly attenuates eccentric contraction injury.

### ILP using rAAV8 in cynamologous macaques results in widespread gene expression in the lower limb

**Figure 6:**
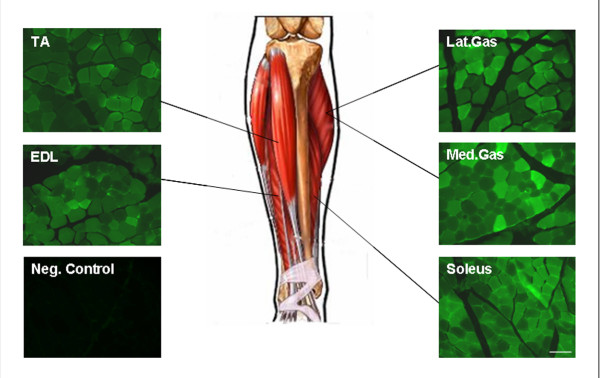
Isolated limb perfusion of rAAV8.CMV.eGFP in cynomologous macaques (4–5 kg) resulted in widespread gene expression in lower limb muscles. Using a custom (PE 50 Tubing) made catheter and the aid of a 0.018 m guide wire and fluoroscopy, 2 × 10^12 ^vg/kg (2 ml volume) was administered via the femoral artery. A tourniquet occluded flow during a 10 minute dwell time to allow vector to bind. Three weeks post transfer, robust transduction was observed in muscles perfused by peroneal vessels (TA and EDL) and tibial vessels (Med and Lat. Gas. – medial and lateral gastrocnemius and soleus) (n = 2). Scale Bar, 100 μm.

The translational goal is to apply pre-clinical findings to a clinical paradigm. Non-human primates (NHP) offer a testing ground to extend vascular delivery to a model with anatomic parallels to humans, as well as to establish approximate dosing ranges similar to a young child. The dose administered to macaques was proportional (based on animal weight) to that given to the mice (5 × 10^12 ^vg/kg in mouse and 2 × 10^12 ^vg/kg in the NHP). The left hindlimb of two cynamologous macaques (4–5 kg animals) were perfused with approximately 10^13 ^vg (2 × 10^12^/kg) of rAAV8.CMV.eGFP in 2 ml PBS using a custom made, fluoroscopy guided catheter that was advanced to the arterial bifurcation at the level of the knee. The procedure mirrored the mouse studies with a pre-flush (2 ml) prior to obstructing flow, a 10 minute dwell time and delivery of a post-flush (2 ml) immediately before removing the tourniquet (although, the femoral vein was not catheterized for this experiment). Three weeks post transfer, the animals were euthanized and the lower limb muscles including the TA, EDL, gastrocnemius and soleus were dissected to compare with mdx studies. GFP was visualized by direct fluorescence and demonstrated widespread gene expression in all muscles analyzed [medial gastrocnemius – 63.8 ± 4.9%, lateral gastrocnemius – 66.0 ± 4.5%, EDL – 80.2 ± 3.1%, soleus – 86.4 ± 1.9%, TA – 72.2 ± 4.0% (Fig. 6). Means were obtained by counting 4 fields from 4–6 muscle blocks for each muscle spanning proximal, central, and distal regions for a total of 16–24 – 20× fields (field = 0.349 mm^2^) per muscle. These data demonstrate that rAAV8 is efficient at gaining access to the underlying musculature in the primate host and appears to be an ideal serotype for delivery to multiple muscle groups via the vasculature using a low volume, low pressure, and low dose experimental paradigm.

## Discussion

Successful gene therapy for DMD will require widespread muscle transduction using a replacement gene, presumably a mini- or micro-dystrophin or a surrogate gene. [20,26] Success in the mdx mouse with tail vein delivery is notable, [9,10] but key issues for translation to clinical trials have been wanting. In this study we focused on a vascular delivery system that can be adapted for a clinical protocol in an effort to achieve a clinically meaningful outcome, providing safe passage for the virus, and using a dose within the manufacturing constraints for rAAV vectors. We reasoned that a regional vascular delivery approach to the lower limbs through the femoral artery could potentially meet these expectations. This is similar in concept to local versus systemic gene transfer to the liver as demonstrated by Hodges et al. [27]

The first step in this process was to identify the appropriate AAV serotype that would efficiently cross the vascular barrier using a conditional paradigm of low pressure and low volume. Based on prior studies, there are advocates for AAV6 and AAV8; however, viral loads, vascular transport enhancing agents, and ages of animals [10,15] preclude direct comparisons between these serotypes in the published literature. AAV1 also has demonstrated activity following vascular delivery in the mouse, [18,25] but again conditions for delivery cannot be directly compared with AAV6 and AAV8. For clinical trials, we anticipate a regional vascular delivery approach. The femoral artery supplying major muscle groups in the lower extremity represents a potential first target necessitating comparing AAV serotypes (AAV1, AAV6, and AAV8), carrying the same transgene (a modified micro-dystrophin), under control of the same promoter (MCK), using identical delivery conditions appropriate for a clinical setting. The results were unambiguous. AAV6 and AAV8 performed equally well for regional vascular delivery to the lower limb, which was strikingly different from AAV1. We found widespread micro-dystrophin expression in the TA and EDL at 1, 2, and 3 months post gene transfer (Fig. 1B), with insignificant differences in quantitation of transduced myofibers (rAAV8 > 90% and rAAV6 > 80%). These transduction efficiencies through a vascular route of delivery demonstrated a significant increase in isometric force generation and partial protection from contraction-induced injury compared to mdx (Fig. 5).

The transduction efficiency for rAAV1 was dramatically lower (<3%). We attribute the differences between our results with AAV1 and those of prior studies [18,25] most likely related to the volume of perfusate [1 ml vs 100 μl (current study) per 20 g body weight] and the rate of administration [delivered over 10–39 seconds vs 60–80 seconds (current study)]. Using AAV1 for vascular gene transfer, Gonin et al. demonstrated capillary rupture within the endomysium that more than likely accounts for the high rate of gene expression. They also observed edema of the extremity. Although we did not directly examine the capillary bed in our preparations, we suggest that vascular integrity was maintained based on the following: 1) there was no extremity swelling in our mice; 2) AAV1, AAV6, and AAV8 demonstrated equal transduction efficiencies following intramuscular injections with >94% gene expression, yet impressive differences were seen following vascular gene transfer suggesting a distinction based on vascular endothelial transport, or 3) preferential inhibitory binding within the vasculature by rAAV1.

In the course of testing the serotypes for delivery of micro-dystrophin it was our intent to develop a gene transfer approach that could potentially be adapted to the clinic. In mouse we used isolated limb perfusion, a pre-flush of saline prior to gene delivery, a dwell time with all circulation occluded, and a post flush with an open venous system to rid the extremity of as much vector as possible. We believe that a pre-flush could have particular significance in a clinical trial where pre-existing antibody is a major threat to organ transduction. [27,28] Even in the absence of pre-existing immunity, delivery of the virus in an environment free of serum and particulate material (complement proteins, platelets, erythrocytes, leucocytes), with its potential viral-binding capacity, provides a clearer path to muscle transduction and an added safety factor because of reduced risk for carrying virus to remote sites. In addition, a prolonged dwell time with occluded circulation in the presence of platelets and other clotting factors could lead to a blood clot in the extremity with potential serious consequences.

In the mouse, attention to the factors outlined resulted in 80% to 90% muscle transduction using rAAV6 and rAAV8 carrying micro-dystrophin. In an attempt to avoid putting too much emphasis on success in the mouse, we extended our studies to the non-human primate employing conditions that would closely simulate a clinical regional vascular delivery model. Thus, in the monkey we directly tested rAAV8 delivery of eGFP through an isolated femoral artery using conditions that would be applicable for a clinical trial (low pressure, low volume, and without pharmacologic agents). We used viral doses appropriate for clinical production runs (2 × 10^12 ^vg/kg), and at the same time achieved levels of muscle gene expression in lower limbs predictive of a therapeutic benefit. [29]

Our study fulfills stringent criteria for gene delivery with implications for clinical application using an approach and AAV serotype that will potentially accelerate efforts to produce life-altering results in children with DMD, with applicability to other muscular dystrophies. Additional work in the monkey is required, especially using a cassette more analogous to one that will be used in practice. Studies in the non-human primate do provide a number of advantages including a >90% genomic sequence homology to humans, accounting for wide application to vaccine research and organ transplantation. The monkey is also well thought of for translational research for gene therapy owing to its high rate of natural infection by AAV similar to humans. [30] This is particularly relevant to studies of AAV8 gene transfer where translational paradigms, using monkeys with and without pre-existing immunity, permit study of specific immunosuppressive regimens. To successfully transfer rAAV to a canine dystrophy model, Wang et al. [19] were required to use anti-thymocyte globulin, cyclosporine and mycophenolate mofetil. The work presented here paves the way for studies of gene expression in a primate host under conditions that will be appropriate for the clinic using regional vascular delivery. Specific muscle groups in the limbs can be perfused and critical sites, like the diaphragm and heart, can be targeted. Studies in the monkey will also permit further sophistication in gene delivery, using fluoroscopy-guided balloon catheters for precise delivery of vector to isolated vascular regions with the advantage of subsequent removal of unbound virus reducing potential for spread to remote sites.

## Competing interests

The author(s) declare that they have no competing interests.

## Authors' contributions

LRK generated the rAAV.MCK.micro-dystrophin construct and the corresponding rAAV1, rAAV6, and rAAV8 vectors, performed intramuscular injections, harvested muscle tissue, and performed all immunohistochemistry and morphometric analysis. PMLJ conducted all physiology experiments. CLM, BDC, LRK, and LGC conducted the isolated limb perfusion experiments in mice and non-human primates. KRC oversaw rAAV vector production. LRK, LGC, KRC, and JRM conceived the study, participated in its design and coordination, and drafted the manuscript. All authors read and approved the final manuscript.

## References

[B1] Emery AE (1991). Population frequencies of inherited neuromuscular diseases--a world survey. Neuromuscul Disord.

[B2] Mendell JR, Moxley RT, Griggs RC, Brooke MH, Fenichel GM, Miller JP, King W, Signore L, Pandya S, Florence J (1989). Randomized, double-blind six-month trial of prednisone in Duchenne's muscular dystrophy. N Engl J Med.

[B3] Aartsma-Rus A, Kaman WE, Weij R, den Dunnen JT, van Ommen GJ, van Deutekom JC (2006). Exploring the frontiers of therapeutic exon skipping for Duchenne muscular dystrophy by double targeting within one or multiple exons. Mol Ther.

[B4] McClorey G, Moulton HM, Iversen PL, Fletcher S, Wilton SD (2006). Antisense oligonucleotide-induced exon skipping restores dystrophin expression in vitro in a canine model of DMD. Gene Ther.

[B5] Barton-Davis ER, Cordier L, Shoturma DI, Leland SE, Sweeney HL (1999). Aminoglycoside antibiotics restore dystrophin function to skeletal muscles of mdx mice. J Clin Invest.

[B6] Hamed SA (2006). Drug evaluation: PTC-124--a potential treatment of cystic fibrosis and Duchenne muscular dystrophy. IDrugs.

[B7] Tinsley J, Deconinck N, Fisher R, Kahn D, Phelps S, Gillis JM, Davies K (1998). Expression of full-length utrophin prevents muscular dystrophy in mdx mice. Nat Med.

[B8] Bogdanovich S, Krag TO, Barton ER, Morris LD, Whittemore LA, Ahima RS, Khurana TS (2002). Functional improvement of dystrophic muscle by myostatin blockade. Nature.

[B9] Gregorevic P, Allen JM, Minami E, Blankinship MJ, Haraguchi M, Meuse L, Finn E, Adams ME, Froehner SC, Murry CE, Chamberlain JS (2006). rAAV6-microdystrophin preserves muscle function and extends lifespan in severely dystrophic mice. Nat Med.

[B10] Gregorevic P, Blankinship MJ, Allen JM, Crawford RW, Meuse L, Miller DG, Russell DW, Chamberlain JS (2004). Systemic delivery of genes to striated muscles using adeno-associated viral vectors. Nat Med.

[B11] Liu M, Yue Y, Harper SQ, Grange RW, Chamberlain JS, Duan D (2005). Adeno-associated virus-mediated microdystrophin expression protects young mdx muscle from contraction-induced injury. Mol Ther.

[B12] Yoshimura M, Sakamoto M, Ikemoto M, Mochizuki Y, Yuasa K, Miyagoe-Suzuki Y, Takeda S (2004). AAV vector-mediated microdystrophin expression in a relatively small percentage of mdx myofibers improved the mdx phenotype. Mol Ther.

[B13] Sampaolesi M, Blot S, D'Antona G, Granger N, Tonlorenzi R, Innocenzi A, Mognol P, Thibaud JL, Galvez BG, Barthelemy I, Perani L, Mantero S, Guttinger M, Pansarasa O, Rinaldi C, Cusella De Angelis MG, Torrente Y, Bordignon C, Bottinelli R, Cossu G (2006). Mesoangioblast stem cells ameliorate muscle function in dystrophic dogs. Nature.

[B14] Cerletti M, Negri T, Cozzi F, Colpo R, Andreetta F, Croci D, Davies KE, Cornelio F, Pozza O, Karpati G, Gilbert R, Mora M (2003). Dystrophic phenotype of canine X-linked muscular dystrophy is mitigated by adenovirus-mediated utrophin gene transfer. Gene Ther.

[B15] Wang Z, Zhu T, Qiao C, Zhou L, Wang B, Zhang J, Chen C, Li J, Xiao X (2005). Adeno-associated virus serotype 8 efficiently delivers genes to muscle and heart. Nat Biotechnol.

[B16] Xiao W, Chirmule N, Berta SC, McCullough B, Gao G, Wilson JM (1999). Gene therapy vectors based on adeno-associated virus type 1. J Virol.

[B17] Schnepp BC, Clark KR, Klemanski DL, Pacak CA, Johnson PR (2003). Genetic fate of recombinant adeno-associated virus vector genomes in muscle. J Virol.

[B18] Goyenvalle A, Vulin A, Fougerousse F, Leturcq F, Kaplan JC, Garcia L, Danos O (2004). Rescue of dystrophic muscle through U7 snRNA-mediated exon skipping. Science.

[B19] Wang Z, Kuhr CS, Allen JM, Blankinship M, Gregorevic P, Chamberlain JS, Tapscott SJ, Storb R (2007). Sustained AAV-mediated dystrophin expression in a canine model of Duchenne muscular dystrophy with a brief course of immunosuppression. Mol Ther.

[B20] Harper SQ, Hauser MA, DelloRusso C, Duan D, Crawford RW, Phelps SF, Harper HA, Robinson AS, Engelhardt JF, Brooks SV, Chamberlain JS (2002). Modular flexibility of dystrophin: implications for gene therapy of Duchenne muscular dystrophy. Nat Med.

[B21] Rabinowitz JE, Rolling F, Li C, Conrath H, Xiao W, Xiao X, Samulski RJ (2002). Cross-packaging of a single adeno-associated virus (AAV) type 2 vector genome into multiple AAV serotypes enables transduction with broad specificity. J Virol.

[B22] Clark KR, Liu X, McGrath JP, Johnson PR (1999). Highly purified recombinant adeno-associated virus vectors are biologically active and free of detectable helper and wild-type viruses. Hum Gene Ther.

[B23] Palmiter RD, Sandgren EP, Avarbock MR, Allen DD, Brinster RL (1991). Heterologous introns can enhance expression of transgenes in mice. Proc Natl Acad Sci U S A.

[B24] Fougerousse F, Bartoli M, Poupiot J, Arandel L, Durand M, Guerchet N, Gicquel E, Danos O, Richard I (2007). Phenotypic Correction of alpha-Sarcoglycan Deficiency by Intra-arterial Injection of a Muscle-specific Serotype 1 rAAV Vector. Mol Ther.

[B25] Gonin P, Arandel L, Van Wittenberghe L, Marais T, Perez N, Danos O (2005). Femoral intra-arterial injection: a tool to deliver and assess recombinant AAV constructs in rodents whole hind limb. J Gene Med.

[B26] Wang B, Li J, Xiao X (2000). Adeno-associated virus vector carrying human minidystrophin genes effectively ameliorates muscular dystrophy in mdx mouse model. Proc Natl Acad Sci U S A.

[B27] Hodges BL, Taylor KM, Chu Q, Scull SE, Serriello RG, Anderson SC, Wang F, Scheule RK (2005). Local delivery of a viral vector mitigates neutralization by antiviral antibodies and results in efficient transduction of rabbit liver. Mol Ther.

[B28] Manno CS, Pierce GF, Arruda VR, Glader B, Ragni M, Rasko JJ, Ozelo MC, Hoots K, Blatt P, Konkle B, Dake M, Kaye R, Razavi M, Zajko A, Zehnder J, Rustagi PK, Nakai H, Chew A, Leonard D, Wright JF, Lessard RR, Sommer JM, Tigges M, Sabatino D, Luk A, Jiang H, Mingozzi F, Couto L, Ertl HC, High KA, Kay MA (2006). Successful transduction of liver in hemophilia by AAV-Factor IX and limitations imposed by the host immune response. Nat Med.

[B29] Foster K, Foster H, Dickson JG (2006). Gene therapy progress and prospects: Duchenne muscular dystrophy. Gene Ther.

[B30] Chirmule N, Propert K, Magosin S, Qian Y, Qian R, Wilson J (1999). Immune responses to adenovirus and adeno-associated virus in humans. Gene Ther.

